# Effects of Enzymatic Cottonseed Protein Concentrate as a Feed Protein Source on the Growth, Plasma Parameters, Liver Antioxidant Capacity and Immune Status of Largemouth Bass (*Micropterus salmoides*)

**DOI:** 10.3390/metabo12121233

**Published:** 2022-12-08

**Authors:** Qile Zhang, Hualiang Liang, Pao Xu, Gangchun Xu, Lu Zhang, Yongli Wang, Mingchun Ren, Xiaoru Chen

**Affiliations:** 1 Wuxi Fisheries College, Nanjing Agricultural University, Wuxi 214081, China; 2 Key Laboratory of Integrated Rice-Fish Farming Ecology, Ministry of Agriculture and Rural Affairs, Freshwater Fisheries Research Center, Chinese Academy of Fishery Sciences, Wuxi 214081, China; 3 Tongwei Co., Ltd., Healthy Aquaculture Key Laboratory of Sichuan Province, Chengdu 610093, China; 4 Tongwei Agricultural Development Co., Ltd., Chengdu 610093, China

**Keywords:** largemouth bass (*Micropterus salmoides*), enzymatic cottonseed protein concentrate, growth performance, antioxidant capacity, immunity

## Abstract

This study appraised the impact of enzymatic cottonseed protein concentrate (ECP) as a fish meal (FM) substitute on the growth and health of largemouth bass (*Micropterus salmoides*) (initial weight 14.99 ± 0.03 g). Five diets with equal nitrogen, fat, and energy were designed to replace 0%, 7.78%, 15.56%, 23.33%, and 31.11% FM by adding 0%, 3.6%, 7.2%, 10.8%, and 14.4% ECP, named ECP0, ECP3.6, ECP7.2, ECP10.8, and ECP14.4, respectively. We fed 300 fish with five experimental diets for 60 days. The results revealed that weight gain rate (WGR) and specific growth rate (SGR) did not notably reduce until the addition of ECP exceeded 7.2%. The proximate composition of fish was not affected by the amount of ECP added in diets. Plasma total protein (TP), albumin (ALB), and high-density lipoprotein (HDL) concentrations increased with the increase of ECP dosage, while the triglyceride (TG) and low-density lipoprotein (LDL) concentrations and alkaline phosphatase (ALP) activity showed an opposite trend. For hepatic antioxidant capacity, the hepatic total superoxide dismutase (T-SOD) and catalase (CAT) activities, glutathione (GSH) content, and the expression of nuclear factor erythroid 2-related factor 2 (Nrf2), superoxide dismutase (SOD), and CAT were increased by ECP, while the hepatic malondialdehyde (MDA) content and the expression of kelch-like-ECH-associated protein 1 (Keap1) were decreased. With regard to inflammation, the expression of nuclear factor-kappa B (NF-κB), interleukin-8 (IL-8), and tumor necrosis factor-α (TNF-α) were inhibited by ECP. In summary, the amount of ECP added to diet can reach 7.2% to replace 15.56% FM without hampering the growth of largemouth bass, and ECP can improve the antioxidant and immune capacity.

## 1. Introduction

Largemouth bass (*Micropterus salmoides*) have many advantages, including strong adaptability, fast growth, no intermuscular spines, and good flavor; they are widely liked by humans and have been largely farmed in many countries [1]. The requirement of largemouth bass for dietary protein is high, given that they are carnivorous fish, accounting for approximately 40–50% of dry matter, and the dietary protein is mainly provided by fish meal (FM) [2,3,4,5]. In aquatic animals, FM is the best and main source of dietary protein; however, FM resources are limited, the output has difficulty meeting the needs of the rapid development of aquaculture, and the price is gradually rising [6,7,8]. Therefore, it is necessary to investigate new high-quality and inexpensive alternative protein sources for FM.

Cottonseed meal, the residue of cottonseed after oil extraction, is rich in protein content and yield, and low in price [9]. A variety of cottonseed meal products can be added to aquatic feeds as protein ingredients [10,11,12,13,14,15,16,17]. The application of cottonseed meal not only avoids wasting cottonseed resources but also alleviates the shortage of feed protein resources. As there are some anti-nutritional factors, cottonseed meal needs to be processed to improve the utilization rate. Among various processing methods, enzymatic hydrolysis is an effective and popular method for improving the quality of plant protein because of its non-irritating reaction conditions, less harmful products, lack of effect on the nutritional value of amino acids, and low pollution level on the environment [18]. Previous studies showed that plant proteins with enzymatic hydrolysis treatment have better application effects in animal feed [19,20]. Enzymatic hydrolysis treatment could increase the water-soluble protein, total amino acids, and peptide fraction contents of cottonseed meal [21,22]. Furthermore, various active peptides, such as antioxidant peptides and antimicrobial peptides, could be produced from the enzymatic hydrolysis of cottonseed protein [23,24,25]. It was reported that crucian carp (*Carassius auratus gibelio*) fed with a diet containing 5% cottonseed meal hydrolysate instead of cottonseed meal had better growth, feed utilization rate, and absorption of zinc and iron [21]. Chinese soft-shelled turtle (*Pelodiscus sinensis*) fed with diets containing cottonseed meal protein hydrolysate had better growth, feed intake, digestive enzymes activity, and intestinal development [26]. In addition, it was reported that adding 3% cottonseed meal protein hydrolysate to the feed of blunt snout bream (*Megalobrama amblycephala*) could reduce FM content from 6% to 2.8%, resulting in better growth performance, antioxidant capacity, and immunity [27].

Up to now, studies on replacing dietary FM with enzymatic cottonseed protein concentrate (ECP) in largemouth bass are still lacking. Therefore, the current study was conducted to investigate the effects of ECP on the growth, proximate composition, plasma biochemical indexes, hepatic antioxidant capacity, and immune status of largemouth bass, and evaluate the potential of ECP as a feed protein material to promote the development of low FM feed for aquatic animals.

## 2. Results

### 2.1. Growth Performance

As displayed in Table 1, fish fed with ECP10.8 and ECP14.4 diets were observed to have lower final weight (FW), weight gain rate (WGR), and specific growth rate (SGR) (*p* > 0.05). Compared with the control diet (ECP0), fish fed with ECP3.6, ECP7.2, ECP10.8, and ECP14.4 diets were observed to have similar feed intake (FI), feed coefficient rate (FCR), protein efficiency ratio (PER), and survival rate (SR) (*p* > 0.05).

### 2.2. Proximate Composition of Whole Fish

As displayed in Table 2, the moisture, crude protein, crude lipid, and ash contents of fish fed with different diets had no significant difference (*p* > 0.05).

### 2.3. Plasma Parameters

As displayed in Table 3, ECP tended to increase the plasma total protein (TP), albumin (ALB), and high-density lipoprotein (HDL) concentrations, while tending to decrease the total cholesterol (TC), triglyceride (TG), and low-density lipoprotein (LDL) concentrations and alkaline phosphatase (ALP) activity. Compared with the control diet, fish fed with ECP7.2, ECP10.8, and ECP14.4 diets had higher TP and ALB concentrations, and fish fed with ECP-containing diets had higher HDL concentrations (*p* < 0.05). Fish fed with ECP14.4 diets had the lowest TC, LDL concentrations, and ALP activity.

### 2.4. Hepatic Antioxidant Parameters and MDA Levels

**Figure 1:**
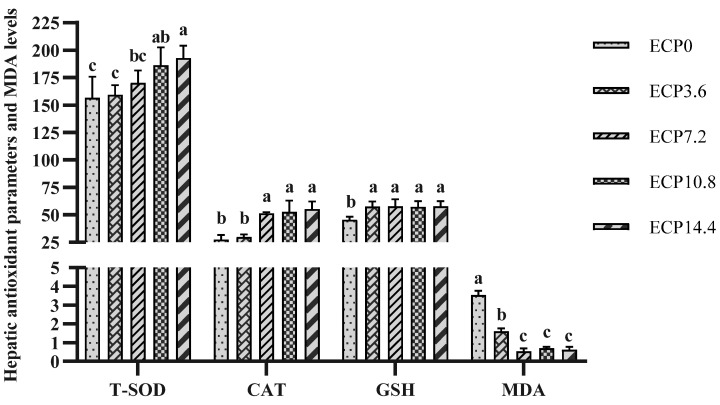
Hepatic antioxidant parameters and MDA levels. T-SOD, total superoxide dismutase; CAT, catalase; GSH, glutathione; MDA, malondialdehyde. Data are presented as mean ± SD. Bars with different superscripts indicate significant differences (*p* < 0.05), while that with the same letter or no letter superscripts indicate no significant differences (*p* > 0.05).

As shown in Figure 1, ECP tended to improve the hepatic antioxidant capacity of largemouth bass. Compared with the control diet, higher total superoxide dismutase (T-SOD) and catalase (CAT) activities were observed in fish fed with ECP10.8 and ECP14.4 diets, and fish fed with ECP7.2, ECP10.8, and ECP14.4 diets, respectively (*p* < 0.05). Fish fed with ECP-containing diets were observed to have higher glutathione (GSH) content and lower malondialdehyde (MDA) content (*p* < 0.05).

### 2.5. Expression of Antioxidant Genes mRNA

**Figure 2:**
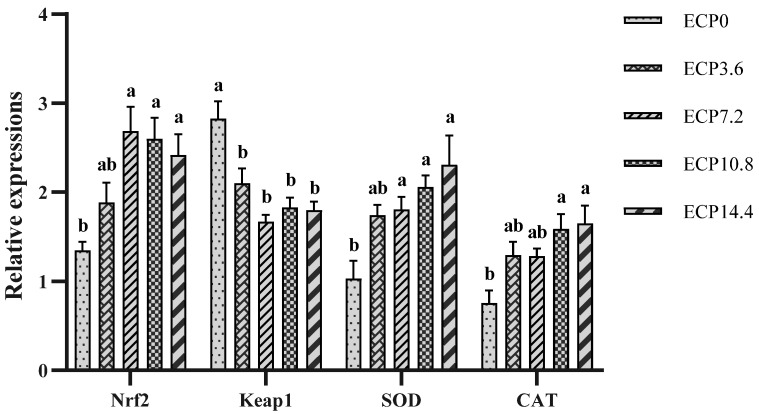
Relative mRNA expression levels of antioxidant genes in liver. Nrf2, nuclear factor erythroid 2-related factor 2; Keap1, kelch-like-ECH-associated protein 1; SOD, superoxide dismutase; CAT, catalase. Data are presented as mean ± SD. Bars with different superscripts indicate significant differences (*p* < 0.05), while that with the same letter or no letter superscripts indicate no significant differences (*p* > 0.05).

As presented in Figure 2, compared with the control diet, ECP7.2, ECP10.8, and ECP14.4 diets significantly increased the mRNA expression level of nuclear factor erythroid 2-related factor 2 (Nrf2) and superoxide dismutase (SOD), ECP10.8 and ECP14.4 diets significantly increased the mRNA expression level of CAT, and ECP-containing diets significantly decreased the mRNA expression level of kelch-like-ECH-associated protein 1 (Keap1) (*p* < 0.05).

### 2.6. Expression of Immune-Related Genes mRNA

**Figure 3:**
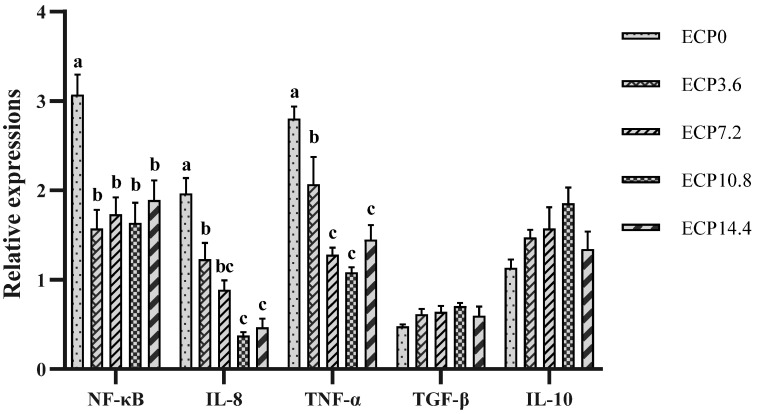
Relative mRNA expression levels of immune-related genes in liver. NF-κB, nuclear factor-kappa B; IL-8, interleukin-8; TNF-α, tumor necrosis factor-α; TGF-β, transforming growth factor-β; IL-10, Interleukin-10. Data are presented as mean ± SD. Bars with different superscripts indicate significant differences (*p* < 0.05), while that with the same letter or no letter superscripts indicate no significant differences (*p* > 0.05).

As presented in Figure 3, compared with the control diet, ECP-containing diets significantly decreased the mRNA expression level of nuclear factor-kappa B (NF-κB), interleukin-8 (IL-8), and tumor necrosis factor-α (TNF-α) (*p* < 0.05). ECP-containing diets tended to increase the mRNA expression level of transforming growth factor-β (TGF-β) and interleukin-10 (IL-10), but not significantly (*p* > 0.05).

## 3. Discussion

As the source of feed protein, various cottonseed protein products have been studied to replace dietary FM in many fish species [10,11,12,13,14,15,16,17]. In the current study, results showed that fish fed with a diet containing 3.6% ECP presented the best growth performance, and 7.2% ECP could be incorporated into the diet to replace 15.56% FM (based on the 45% FM group) without reducing growth performance. However, the growth performance was markedly decreased once the amount of ECP was increased to 10.8% and 14.4%. Likewise, the substitution of cottonseed meal protein hydrolysate for FM had similar effects on blunt snout bream [27]. Similar effects of replacing FM with plant protein hydrolysates have also been found in turbot (*Scophthalmus maximus*), rainbow trout, and largemouth bass [28,29,30]. The good nutritional value of protein hydrolysates may be the reason for the successful substitution of partial FM. A study on Atlantic cod (*Gadus morhua*) reported that feeding an intact protein diet led to the retaining of more nutrients than feeding an amino acid diet, although the amino acid diet showed faster absorption [31]. The rapid absorption of amino acids and peptides may accelerate the excretion of amino acids or lead to the imbalance of the intake of amino acids, thus reducing the utilization of the amino acid diet. The cottonseed protein concentrate used in the current study was hydrolyzed into amino acids and peptides by enzymes. Once the amount of ECP in the diet was too high, the amino acids and peptides ingested by fish might be supersaturated, leading to a decline in the utilization of ECP, which may be a reason for the decline in growth. Moreover, the addition of ECP notably depressed the activity of plasma ALP in the current study. ALP catalyzes the phosphorylation of many types of molecules under alkaline pH conditions and is involved in membrane transport activities and mineralization of the fish skeleton [32,33]. Therefore, the low activity of ALP possibly contributes to the weak growth performance. The specific reasons for the decline in the growth performance of largemouth bass caused by the addition of ECP to diet need to be confirmed by further research.

In the current study, the proximate composition of fish fed with different diets had no distinct difference, indicating that ECP has little effect on the proximate composition of largemouth bass. Similarly, the substitution of cottonseed meal protein hydrolysate for FM did not affect the proximate composition of blunt snout bream [27]. However, previous studies obtained different results, which indicated that cottonseed protein concentrates in diets affected the proximate composition of largemouth bass [34,35,36,37]. The diversity of cottonseed protein products may be the main reason for these different results [38].

Plasma TP and ALB are related to the immune status of fish [39,40]. Results from the current study showed that replacing FM with ECP increased the plasma TP and ALB concentrations, indicating that ECP may enhance the immune response of largemouth bass. Similarly, the substitution of cottonseed meal protein hydrolysate for FM increased the plasma TP and ALB concentrations of blunt snout bream [27]. Diets supplemented with protein hydrolysates also increased the plasma protein level of Olive flounder (*Paralichthys olivaceus*) [41]. To avoid cardiovascular disease caused by lipid accumulation, it is considered to be beneficial when the content of TC, TG, and LDL in plasma are low and the HDL content is high [42,43]. Results from the current study showed that replacing FM with ECP increased plasma HDL concentrations, while decreasing TC, TG, and LDL concentrations, suggesting that ECP might have the effect of lowering plasma lipids and preventing cardiovascular disease. A study on starry flounder (*Platichthys stellatus*) also found that replacing FM with plant protein hydrolysates reduced plasma lipid levels [44].

Antioxidant defense systems, including enzymatic and nonenzymatic systems, play an important role in maintaining fish health [45,46]. MDA, one of the final products of cell membrane lipid peroxidation, is a commonly used indicator to measure the degree of oxidative stress [47]. Results from the current study revealed that the substitution of ECP for FM had a positive effect on increasing hepatic T-SOD and CAT activities and the GSH content while reducing the hepatic MDA content. Correspondingly, results of mRNA expression of antioxidant genes revealed that ECP upregulated the mRNA expression level of Nrf2, SOD, and CAT. These results indicate that ECP could activate the Nrf2 signaling pathway and improve hepatic antioxidant enzyme activity to reduce the oxidative stress of largemouth bass. Similarly, the antioxidant capacities and innate immunity of blunt snout bream hepatocytes were improved by cottonseed meal protein hydrolysate [48]. The antioxidant effect may be due to the fact that ECP contains antioxidant peptides. It was reported that peptide fractions derived from enzymatically hydrolyzed cottonseed protein had obvious effects on inhibiting the formation of MDA in the linoleic acid autoxidation system and scavenging various free radicals [49]. Furthermore, it was reported that the antioxidant capacity of peptides from cottonseed protein hydrolysates remained high or was even enhanced after in vitro digestion [50]. However, there are some different findings, for instance, the hepatic antioxidant capacity of Ussuri catfish (*Pseudobagrus ussuriensis*) was decreased by the substitution of cottonseed meal for dietary FM [51]. Both the serum antioxidant enzyme activities and MDA content in hybrid grouper (♀*Epinephelus fuscoguttatus* × ♂*Epinephelus lanceolatu*) were increased by the substitution of cottonseed protein concentrate for dietary FM [11]. Different results are possibly attributed to the specificity of fish species and tissue, as well as differences in the methods for producing cottonseed protein products [52]. It was reported that, compared with soybean meal or soy protein concentrate, enzyme-treated soybean meal had a more effective role in improving the antioxidant capacity [19].

In addition to the antioxidant system, the liver immune state is intimately related to fish health and growth. It was reported that NF-κB, the critical regulator of proinflammatory gene expression, plays a significant role in inflammation [53]. In the current study, ECP instead of FM markedly inhibited the mRNA expression of NF-κB and proinflammatory cytokines (IL-8 and TNF-α), while it tended to promote the mRNA expression of anti-inflammatory cytokines (TGF-β and IL-10), indicating that ECP has the function of restraining inflammation by inhibiting the NF-κB signal pathway. It was demonstrated that oxidative stress is related to the pathogenesis of various inflammatory diseases, and antioxidant agents can mitigate inflammation [54]. Therefore, the antioxidant peptides produced by the enzymatic hydrolysis of cottonseed protein can not only alleviate oxidative stress but also contribute to suppressing inflammation. Conversely, previous studies reported that cottonseed protein instead of FM induced the expression of proinflammatory genes while suppressing the expression of anti-inflammatory genes in hybrid grouper and silver sillago (*Sillago sihama Forsskál*) [11,55]. Compared with cottonseed protein concentrate, ECP may better improve antioxidant performance and immunity [34,35]. The diversity between the results of this study and previous studies is likely due to the different cottonseed protein treatment methods. It was reported that, compared with conventional dehulled soybean meal, soybean meal that has undergone enzyme treatment processing effectively improved the nonspecific immunity of largemouth bass [20]. Hence, enzymatic hydrolysis is a great processing method to improve cottonseed protein quality.

## 4. Materials and Methods

### 4.1. Experimental Diets

We replaced 0%, 7.78%, 15.56%, 23.33%, and 31.11% FM with 0%, 3.6%, 7.2%, 10.8%, and 14.4% ECP to formulate five iso-nitrogenous (49%) and iso-energetic (19 kJ/g) experimental diets, named ECP0, ECP3.6, ECP7.2, ECP10.8, and ECP14.4, respectively (Table 4). The steps to produce the experimental diets included crushing the ingredients and passing them through 80 mesh sieves, weighing the ingredients according to the formula, fully mixing various ingredients, and granulating the diet using a pelletizer (F-26 (II), South China University of Technology, China). The pellet diets were dried in a ventilated oven at 45 ℃, then bagged and placed at −20 °C until use.

### 4.2. Feeding Trial

The feeding trial was conducted at the Charoen Pokphand Group breeding farm (Huanggang, Hubei, China). Experiment fish were purchased from the Yongda Aquaculture Professional Cooperative (Ezhou, Hubei, China). Firstly, fish were fed with a commercial diet twice a day for two weeks to acclimate to the experimental conditions. We fasted fish for 24 h, then selected 300 lively fish (average initial weight 14.99 ± 0.03 g) and randomly put them into 15 cages (1 m × 1 m × 1 m), with 20 fish per cage and 3 cages per group. The fish were fed with experimental diets to apparent satiety two times (6:30 and 18:30) every day; the feeding trial lasted for 60 days. During the trial period, the water temperature, pH, ammonia nitrogen content, nitrite content, and dissolved oxygen concentration were 28–31 °C, 7.5–8.2, 0–0.2 mg/L, 0.1–0.3 mg/L, and ≥6 mg/L, respectively.

### 4.3. Sample Collection

The fish were kept in starvation for 24 h before sampling. Then, we collected the quantity and weight of fish in each cage to calculate the growth indicators. Two fish were collected from each cage for general composition analysis, and three fish from each cage were collected for obtaining blood and liver samples. Sample fish were anesthetized with MS-222 before collecting the blood and liver samples. Blood was collected from the tail vein, then centrifuged in a centrifuge at a speed of 3500 rpm for 10 min to collect the upper plasma. The fish was dissected immediately after blood collection to collect liver samples. Serum samples and liver samples were stored at −80 °C.

### 4.4. Experimental Parameter Detection

The proximate composition analyses of diets and fish were conducted following the methods of AOAC (2003) [56]. We measured the plasma biochemical parameters on an automatic biochemical analyzer with related assay kits. We determined the hepatic antioxidant parameters and MDA levels through the corresponding assay kit. The main methods, assay kits, and testing equipment for index detection are shown in Table 5.

The measurement of relative mRNA expression included extracting total RNA from tissues, detecting the concentration and quality of RNA, and performing quantitative real-time PCR analysis. More detailed information is presented in our previous study [57]. Glyceraldehyde-3-phosphate dehydrogenase (GAPDH) was chosen as the reference gene, and its expression in different groups was shown to be stable [57]. Pfaffl’s model was used to analyze the gene expression levels [58]. The specific primer sequences used in this study were designed by reference to previous studies [59,60,61,62] and are displayed in Table 6.

### 4.5. Statistical Analysis

One-way ANOVA in SPSS 26.0 software was used for the statistical analysis of experimental data. All experimental data were confirmed to conform to normal distribution and homogeneity of variance before any statistical analysis. All data were displayed as mean ± SD. *p* < 0.05 indicated that the variables in different groups were significantly different, and Tukey’s multiple comparisons were conducted.

## 5. Conclusions

The results from the present study showed that 7.2% ECP could be incorporated into the diet to replace 15.56% FM (based on the 45% FM group) without affecting the growth performance of juvenile largemouth bass. ECP-containing diets can improve the hepatic antioxidant capacity and immunity of largemouth bass.

## Figures and Tables

**Table 1:** Effect of replacing FM with ECP on growth performance of juvenile largemouth bass (*Micropterus salmoides*) ^1^.

Diets	IW (g) ^2^	FW (g) ^3^	WGR (%) ^4^	SGR (%/day) ^5^	FI (g/fish) ^6^	FCR ^7^	PER ^8^	SR (%) ^9^
ECP0	14.88 ± 0.06	72.82 ± 2.66 ^ab^	389.32 ± 19.31 ^a^	2.65 ± 0.07 ^a^	72.09 ± 1.15 ^ab^	1.26 ± 0.06	1.59 ± 0.08	95.00 ± 5.00
ECP3.6	15.03 ± 0.12	76.77 ± 0.86 ^a^	411.75 ± 0.88 ^a^	2.72 ± 0.00 ^a^	70.95 ± 2.94 ^ab^	1.25 ± 0.10	1.61 ± 0.12	91.67 ± 5.77
ECP7.2	15.00 ± 0.13	71.11 ± 0.61 ^b^	374.12 ± 8.08 ^a^	2.59 ± 0.03 ^a^	73.41 ± 1.33 ^a^	1.33 ± 0.05	1.52 ± 0.05	95.00 ± 8.66
ECP10.8	15.08 ± 0.03	60.78 ± 0.75 ^c^	303.20 ± 5.93 ^b^	2.33 ± 0.02 ^b^	66.90 ± 2.21 ^b^	1.37 ± 0.14	1.46 ± 0.15	98.33 ± 2.89
ECP14.4	14.97 ± 0.18	64.22 ± 2.07 ^c^	329.22 ± 16.57 ^b^	2.43 ± 0.07 ^b^	70.20 ± 2.08 ^ab^	1.46 ± 0.02	1.37 ± 0.02	88.33 ± 5.77

^1^ Data are presented as mean ± SD. ^2^ Initial body weight (IW). ^3^ Final body weight (FW). ^4^ Weight gain rate (WGR, %) = 100 × (final weight (g) − initial weight (g))/initial weight (g). ^5^ Specific growth rate (SGR, %/day) = 100 × ((Ln (final body weight (g)) − Ln (initial body weight (g)))/days). ^6^ Feed intake (FI, g/fish) = dry feed fed (g)/fish number. ^7^ Feed coefficient rate (FCR) = dry feed fed (g)/(final body weight (g) − initial body weight (g)). ^8^ Protein efficiency ratio (PER) = ((final body weight (g) − initial body weight (g))/protein intake (g). ^9^ Survival rate (SR, %) = 100 × (final amount of fish)/(initial amount of fish). ^a–c^ Values in the same column with different superscripts indicate significant differences (*p* < 0.05), while that with the same letter or no letter superscripts indicate no significant differences (*p* > 0.05).

**Table 2:** Effect of replacing FM with ECP on fish whole-body composition of juvenile largemouth bass (*Micropterus salmoides*) ^1^.

Diets	Moisture (%)	Crude Protein (%)	Crude Lipid (%)	Ash (%)
ECP0	67.53 ± 1.46	17.52 ± 0.01	7.95 ± 0.73	4.62 ± 0.19
ECP3.6	67.40 ± 1.04	17.73 ± 0.47	8.55 ± 0.94	4.55 ± 0.36
ECP7.2	67.74 ± 1.01	17.64 ± 0.26	8.06 ± 0.34	4.55 ± 0.16
ECP10.8	68.59 ± 0.27	17.79 ± 0.06	8.07 ± 0.45	4.95 ± 0.23
ECP14.4	66.33 ± 1.57	18.68 ± 0.18	8.97 ± 0.21	4.89 ± 0.27

^1^ Data are presented as mean ± SD.

**Table 3:** Effect of replacing FM with ECP on plasma biochemical parameters of juvenile largemouth bass (*Micropterus salmoides*) ^1^.

Diets	TP (g/L) ^2^	ALB (g/L) ^2^	TC (mmol/L) ^2^	TG (mmol/L) ^2^	LDL (mmol/L) ^2^	HDL (mmol/L) ^2^	ALP (U/L) ^2^
ECP0	55.72 ± 7.21 ^c^	13.61 ± 2.10 ^c^	12.13 ± 2.24	10.00 ± 1.97 ^ab^	4.91 ± 1.60 ^a^	2.37 ± 0.28 ^c^	194.87 ± 52.78 ^a^
ECP3.6	61.48 ± 4.68 ^bc^	15.62 ± 1.81 ^bc^	12.02 ± 1.45	9.56 ± 1.23 ^ab^	4.63 ± 0.87 ^ab^	2.76 ± 0.22 ^b^	161.16 ± 35.10 ^abc^
ECP7.2	69.72 ± 4.04 ^a^	17.34 ± 1.14 ^a^	12.55 ± 1.06	10.97 ± 1.29 ^a^	4.78 ± 0.68 ^a^	3.01 ± 0.13 ^ab^	169.93 ± 31.01 ^ab^
ECP10.8	67.74 ± 5.98 ^ab^	17.42 ± 1.39 ^ab^	11.63 ± 1.35	8.20 ± 0.92 ^b^	3.92 ± 0.83 ^ab^	3.18 ± 0.38 ^a^	143.72 ± 19.23 ^bc^
ECP14.4	67.10 ± 4.30 ^ab^	17.92 ± 1.54 ^a^	10.66 ± 1.65	6.09 ± 1.20 ^c^	3.24 ± 1.17 ^b^	2.96 ± 0.12 ^ab^	119.13 ± 32.61 ^c^

^1^ Data are presented as mean ± SD. ^2^ TP, total protein; ALB, albumin; TC, total cholesterol; TG, triglyceride; LDL, low-density lipoprotein; HDL, high-density lipoprotein; ALP, alkaline phosphatase. ^a–c^ Values in the same column with different superscripts indicate significant differences (*p* < 0.05), while that with the same letter or no letter superscripts indicate no significant differences (*p* > 0.05).

**Table 4:** Ingredients and nutrient composition of experimental diets (% dry basis).

Ingredients (%)	Diets				
	ECP0	ECP3.6	ECP7.2	ECP10.8	ECP14.4
Fish meal ^1^	45.00	41.50	38.00	34.50	31.00
Enzymatic cottonseed protein concentrate ^1^	0.00	3.60	7.20	10.80	14.40
Blood meal ^1^	2.00	2.00	2.00	2.00	2.00
Soybean meal ^1^	13.00	13.00	13.00	13.00	13.00
Corn gluten meal^1^	3.00	3.00	3.00	3.00	3.00
Enzymatic hydrolysis of chicken powder ^1^	4.00	4.00	4.00	4.00	4.00
Wheat meal	7.00	7.00	7.00	7.00	7.00
Cassava starch	7.00	7.00	7.00	7.00	7.00
Rice bran	6.13	6.13	6.13	6.13	6.13
Microcrystalline cellulose	3.27	2.41	1.58	0.82	0.00
Squid Ointment	2.00	2.00	2.00	2.00	2.00
Fish oil	4.10	4.45	4.75	5.00	5.30
Mineral premix ^2^	1.00	1.00	1.00	1.00	1.00
Vitamin premix ^2^	1.00	1.00	1.00	1.00	1.00
Monocalcium phosphate	1.00	1.30	1.60	1.90	2.20
Choline chloride	0.50	0.50	0.50	0.50	0.50
L-Lysine ^3^	0.00	0.08	0.17	0.25	0.33
L-Methionine ^3^	0.00	0.03	0.07	0.10	0.14
Taurine (mg/kg)	0.00	13.30	26.60	39.90	53.20
Proximate Composition (dry basis)					
Crude protein (%)	48.59	48.53	48.61	48.47	48.49
Crude lipid (%)	9.25	9.24	8.98	9.28	9.23
Gross energy (KJ/g)	19.75	19.83	19.87	19.72	19.58

^1^ Fish meal, crude protein 66.73%, crude lipid 9.46%; Enzymatic cottonseed protein concentrate, crude protein 64.85%, crude lipid 0.87%; Blood meal, crude protein 90.68%; Soybean meal, crude protein 53.26%, crude lipid 4.25%; Corn gluten meal, crude protein 59.24%, crude lipid 3.30%; Enzymatically hydrolyzed chicken powder, crude protein 84.62%, crude lipid 1.00%. These materials were obtained from Wuxi Tongwei feedstuffs Co., Ltd., Wuxi, China. ^2^ Mineral premix and vitamins premix were obtained from Wuxi Hanove animal health products Co., Ltd., Wuxi, China. ^3^ The limiting amino acids (L-*lysine* and L-*methionine*) were supplemented according to the levels of the first group.

**Table 5:** The methods and testing equipment of chemical analysis.

Items	Methods, Assay Kits and Testing Equipment
Crude protein	Determined by Hanon K1100 auto kieldahl apparatus (Jinan Hanon Instruments Co., Ltd., Jinan, China).
Crude lipid	Determined by Hanon SOX606 auto fat analy (Jinan Hanon Instruments Co., Ltd., Jinan, China).
Ash	Determined by burning at 550 ℃ for 5 h in a XL-2A intelligent muffle furnace (Hangzhou Zhuochi Instruments Co., Ltd., Hangzhou, China).
Gross energy	Measured by an oxygen bomb calorimeter IKA C6000 ((IKA Works Guangzhou, Guangzhou, China).
TP ^1^	All plasma parameters were determined by Assay kits (Mindray Bio Medical Co., Ltd., Shenzhen, China) with a Mindray BS-400 automatic biochemical analyzer (Mindray Medical International Ltd., Shenzhen, China).
ALB ^1^	
TC ^1^	
TG ^1^	
LDL ^1^	
HDL ^1^	
ALP ^1^	
T-SOD ^2^	All hepatic antioxidant parameters and MDA levels were tested according to the instructions of assay kits purchased from Jian Cheng Bioengineering Institute (Nanjing, China).
CAT ^2^	
GSH ^2^	
MDA ^2^	

^1^ TP, total protein; ALB, albumin; TC, total cholesterol; TG, triglyceride; LDL, low-density lipoprotein; HDL, high-density lipoprotein; ALP, alkaline phosphatase. ^2^ T-SOD, total superoxide dismutase; CAT, catalase; GSH, glutathione; MDA, malondialdehyde.

**Table 6:** Primer sequence for RT-qPCR.

Gene ^1^	Forward Sequence (5′-3′)	Reverse Sequence (5′-3′)	Source
Nrf2	CTGGTCCGAGACATACGC	CTCAGCAGACGCTCCTTC	Zhao et al. (2021) [59]
Keap1	CGTACGTCCAGGCCTTACTC	TGACGGAAATAACCCCCTGC	Yu et al. (2022) [60]
SOD	TGGCAAGAACAAGAACCACA	CCTCTGATTTCTCCTGTCACC	Gu et al. (2022) [61]
CAT	CTATGGCTCTCACACCTTC	TCCTCTACTGGCAGATTCT	Gu et al. (2022) [61]
NF-κB	CCACTCAGGTGTTGGAGCTT	TCCAGAGCACGACACACTTC	Yu et al. (2022) [60]
IL-8	CGTTGAACAGACTGGGAGAGATG	AGTGGGATGGCTTCATTATCTTGT	Yang et al. (2020) [62]
TNF-α	CTTCGTCTACAGCCAGGCATCG	TTTGGCACACCGACCTCACC	Gu et al. (2022) [61]
TGF-β	GCTCAAAGAGAGCGAGGATG	TCCTCTACCATTCGCAATCC	Gu et al. (2022) [61]
IL-10	CGGCACAGAAATCCCAGAGC	CAGCAGGCTCACAAAATAAACATCT	Gu et al. (2022) [61]
GAPDH	ACTGTCACTCCTCCATCTT	CACGGTTGCTGTATCCAA	Yu et al. (2022) [60]

^1^ Nrf2, nuclear factor erythroid 2-related factor 2; Keap1, kelch-like-ECH-associated protein 1; SOD, superoxide dismutase; CAT, catalase; NF-κB, nuclear factor-kappa B; IL-8, interleukin 8; TNF-α, tumor necrosis factor-α; TGF-β, transforming growth factor β; IL-10, interleukin 10; GAPDH, glyceraldehyde-3-phosphate dehydrogenase.

## Data Availability

The authors confirm that the data supporting the findings of this study are available within the manuscript, Tables and Figures.

## References

[1] Hussein G.H.G., Chen M., Qi P.P., Cui Q.K., Yu Y., Hu W.H., Tian Y., Fan Q.X., Gao Z.X., Feng M.W. (2020). Aquaculture industry development, annual price analysis and out-of-season spawning in largemouth bass *Micropterus salmoides*. Aquaculture.

[2] Tidwell J.H., Webster C.D., Coyle S.D. (1996). Effects of dietary protein level on second year growth and water quality for largemouth bass (*Micropterus salmoides*) raised in ponds. Aquaculture.

[3] Huang D., Wu Y.B., Lin Y.Y. (2017). Dietary protein and lipid requirements for juvenile largemouth bass, *Micropterus salmoides*. J. World Aquac. Soc..

[4] Cai Z.N., Qian X.Q., Xie S.Q. (2020). Optimal dietary protein concentrations for largemouth bass (*Micropterus salmoides*) of different sizes (10–500 g). Aquac. Int..

[5] Li X.Y., Zheng S.X., Ma X.K., Cheng K.M., Wu G.Y. (2020). Effects of dietary protein and lipid levels on the growth performance, feed utilization, and liver histology of largemouth bass (*Micropterus salmoides*). Amino Acids.

[6] Hardy R.W. (2010). Utilization of plant proteins in fish diets: Effects of global demand and supplies of fishmeal. Aquac. Res..

[7] Olsen R.L., Hasan M.R. (2012). A limited supply of fishmeal: Impact on future increases in global aquaculture production. Trends Food Sci. Technol..

[8] Jannathulla R., Rajaram V., Kalanjiam R., Ambasankar K., Muralidhar M., Dayal J.S. (2019). Fishmeal availability in the scenarios of climate change: Inevitability of fishmeal replacement in aquafeeds and approaches for the utilization of plant protein sources. Aquac. Res..

[9] Kumar M., Potkule J., Patil S., Saxena S., Patil P.G., Mageshwaran V., Puniad S., Varghesee E., Mahapatra A., Ashtaputrea N. (2021). Extraction of ultra-low gossypol protein from cottonseed: Characterization based on antioxidant activity, structural morphology and functional group analysis. LWT.

[10] Anderson A.D., Alam M.S., Watanabe W.O., Carroll P.M., Wedegaertner T.C., Dowd M.K. (2016). Full replacement of menhaden fish meal protein by low-gossypol cottonseed flour protein in the diet of juvenile black sea bass *Cent*. Striata. Aquac..

[11] Yin B., Liu H.G., Tan B.P., Dong X.H., Chi S.Y., Yang Q.H., Zhang S., Chen L.Q. (2018). Cottonseed protein concentrate (CPC) suppresses immune function in different intestinal segments of hybrid grouper ♀*Epinephelus fuscoguttatus* × ♂*Epinephelus lanceolatu* via TLR-2/MyD88 signaling pathways. Fish Shellfish Immunol..

[12] Wan M.G., Yin P., Fang W.P., Xie S.W., Chen S.J., Tian L.X., Niu J. (2018). The effect of replacement of fishmeal by concentrated dephenolization cottonseed protein on the growth, body composition, haemolymph indexes and haematological enzyme activities of the Pacific white shrimp (*Litopenaeus vannamei*). Aquac. Nutr..

[13] Shen J.F., Liu H.Y., Tan B.P., Dong X.H., Yang Q.H., Chi S.Y., Zhang S. (2020). Effects of replacement of fishmeal with cottonseed protein concentrate on the growth, intestinal microflora, haematological and antioxidant indices of juvenile golden pompano (*Trachinotus ovatus*). Aquac. Nutr..

[14] Ye G.L., Dong X.H., Yang Q.H., Chi S.Y., Liu H.Y., Zhang H.T., Tan B.P., Zhang S. (2020). Low-gossypol cottonseed protein concentrate used as a replacement of fish meal for juvenile hybrid grouper (*Epinephelus fuscoguttatus* ♀ × *Epinephelus lanceolatus* ♂): Effects on growth performance, immune responses and intestinal microbiota. Aquaculture.

[15] Jiang S., Chen Z.B., Zhou F.L., Yang Q.B., Huang J.H., Yang L.S., Li Y.D., Jiang S.G. (2021). Study on partial replacement of fish meal with concentrated dephenolized cottonseed protein in feed of Penaeus monodon. Aquac. Res..

[16] He Y.F., Guo X.W., Tan B.P., Dong X.H., Yang Q.H., Liu H., Zhang S., Chi S.Y. (2021). Replacing fishmeal with cottonseed protein concentrate in feed for pearl gentian groupers (*Epinephelus fuscoguttatus*♀ × *E. lanceolatus*♂): Effects on growth and expressions of key genes involved in appetite and hepatic glucose and lipid metabolism. Aquac. Rep..

[17] Zhao W., Liu Z.L., Niu J. (2021). Growth performance, intestinal histomorphology, body composition, hematological and antioxidant parameters of *Oncorhynchus mykiss* were not detrimentally affected by replacement of fish meal with concentrated dephenolization cottonseed protein. Aquac. Rep..

[18] Sun X.D. (2011). Enzymatic hydrolysis of soy proteins and the hydrolysates utilisation. Int. J. Food Sci. Technol..

[19] Ma X.K., Shang Q.H., Hu J.X., Liu H.S., Brøkner C., Piao X.S. (2019). Effects of replacing soybean meal, soy protein concentrate, fermented soybean meal or fish meal with enzyme-treated soybean meal on growth performance, nutrient digestibility, antioxidant capacity, immunity and intestinal morphology in weaned pigs. Livest. Sci..

[20] Li S.L., Ding G.T., Song F., Sang C.Y., Wang A., Chen N.S. (2020). Comparison of dehulled, fermented and enzyme-treated soybean meal in diets for largemouth bass, *Micropterus salmoides*: Effects on growth performance, feed utilization, immune response and intestinal morphology. Anim. Feed Sci. Technol..

[21] Gui D., Liu W.B., Shao X.P., Xu W.N. (2010). Effects of different dietary levels of cottonseed meal protein hydrolysate on growth, digestibility, body composition and serum biochemical indices in crucian carp (*Carassius auratus gibelio*). Anim. Feed Sci. Technol..

[22] Tang X.P., Xiang R., Chen S.J., Yang S.F., Liu H., Fang R.J., Li A.K. (2018). Effects of Fermented Cottonseed Meal and Enzymatic Hydrolyzed Cottonseed Meal on Amino Acid Digestibility and Metabolic Energy in White Leghorn Rooster. Pak. J. Zool..

[23] Song W.G., Kong X.Z., Hua Y.F., Chen Y.M., Zhang C.M., Chen Y.X. (2020). Identification of antibacterial peptides generated from enzymatic hydrolysis of cottonseed proteins. LWT.

[24] Wang L.Y., Ma M.G., Yu Z.P., Du S.K. (2021). Preparation and identification of antioxidant peptides from cottonseed proteins. Food Chem..

[25] Filho J.G.D.O., Rodrigues J.M., Valadares A.C.F., Almeida A.B.D., Valencia-Mejia E., Fernandes K.F., Lemes A.C., Egea M.B., Dyszy F.H. (2021). Bioactive properties of protein hydrolysate of cottonseed byproduct: Antioxidant, antimicrobial, and angiotensin-converting enzyme (ACE) inhibitory activities. Waste Biomass Valorization.

[26] Wang K.Z., Xu W.N., Zhou M., Zhang D.D., Sun C.X., Qian Y., Liu W.B. (2018). Effects of fishmeal replacement with cottonseed meal protein hydrolysate on growth, digestion and intestinal histology of juvenile Chinese soft-shelled turtle, *Pelodiscus sinensis*. Aquac. Nutr..

[27] Yuan X.Y., Jiang G.Z., Cheng H.H., Cao X.F., Shi H.J., Liu W.B. (2019). An evaluation of replacing fish meal with cottonseed meal protein hydrolysate in diet for juvenile blunt snout bream (*Megalobrama amblycephala*): Growth, antioxidant, innate immunity and disease resistance. Aquac. Nutr..

[28] Song Z.D., Li P.Y., Wang J.Y., Sun Y.Z., Wang C.Q. (2018). Dietary inclusion of hydrolyzed soybean and cottonseed meals influence digestion, metabolic enzymes, and growth-related hormones and growth of juvenile turbot (*Scophthalmus maximus*). Aquac. Int..

[29] Haghbayan S., Shamsaie Mehrgan M. (2015). The effect of replacing fish meal in the diet with enzyme-treated soybean meal (HP310) on growth and body composition of rainbow trout fry. Molecules.

[30] Liu X., Chi S.Y., Li S., Cheng X.L., Gao W.H., Xu Q.Q., Zhang W.B., Zhou X.Q. (2021). Substitution of fish meal with enzyme-treated soybean in diets for juvenile largemouth bass (*Micropterus salmoides*). Aquac. Nutr..

[31] Berge G.E., Lied E., Espe M. (1994). Absorption and incorporation of dietary free and protein bound (U14C)-lysine in Atlantic cod (*Gadus morhua*). Comp. Biochem. Physiol. Part A Physiol..

[32] Öner M., Atli G., Canli M. (2008). Changes in serum biochemical parameters of freshwater fish *Oreochromis niloticus* following prolonged metal (Ag, Cd, Cr, Cu, Zn) exposures. Environ. Toxicol. Chem. Int. J..

[33] Ren M.C., Liu B., Habte-Tsion H.M., Ge X.P., Xie J., Zhou Q.L., Liang H.L., Zhao Z.X., Pan L.K. (2015). Dietary phenylalanine requirement and tyrosine replacement value for phenylalanine of juvenile blunt snout bream, *Megalobrama amblycephala*. Aquaculture.

[34] Liu Y.L., Lu Q.S., Xi L.W., Gong Y.L., Su J.Z., Han D., Zhang Z.M., Liu H.K., Jin J.Y., Yang Y.X. (2021). Effects of replacement of dietary fishmeal by cottonseed protein concentrate on growth performance, liver health, and intestinal histology of largemouth bass (*Micropterus salmoides*). Front. Physiol..

[35] He G.Z., Zhang T.T., Zhou X.M., Liu X.P., Sun H., Chen Y.J., Tan B.P., Lin S.M. (2022). Effects of cottonseed protein concentrate on growth performance, hepatic function and intestinal health in juvenile largemouth bass, *Micropterus salmoides*. Aquac. Rep..

[36] Xu X.Y., Yang H., Zhang C.Y., Bian Y.H., Yao W.X., Xu Z., Wang Y.Y., Li X.Q., Leng X.J. (2022). Effects of replacing fishmeal with cottonseed protein concentrate on growth performance, flesh quality and gossypol deposition of largemouth bass (*Micropterus salmoides*). Aquaculture.

[37] Xie X.Z., Wang J., Guan Y., Xing S.J., Liang X.F., Xue M., Wang J.J., Chang Y., Leclercq E. (2022). Cottonseed protein concentrate as fishmeal alternative for largemouth bass (*Micropterus salmoides*) supplemented a yeast-based paraprobiotic: Effects on growth performance, gut health and microbiome. Aquaculture.

[38] Bian F., Zhou H., He G., Wang C., Peng H., Pu X., Jiang X., Wang X., Mai K. (2017). Effects of replacing fishmeal with different cottonseed meals on growth, feed utilization, haematological indexes, intestinal and liver morphology of juvenile turbot (*Scophthalmus maximus* L.). Aquac. Nutr..

[39] Soyingbe A.A., Ogunyanwo O.O., Hammed T.B., Adesope A.O. (2012). Effects of sublethal concentrations of diazinon on total protein in tilapia fish (*Oreochromis niloticus*). IOSR J. Environ. Sci. Toxicol. Food Technol..

[40] Ren M.C., Liang H.L., He J., Masagounder K., Yue Y., Yang H., Ge X.P., Xi B.W. (2017). Effects of DL-methionine supplementation on the success of fish meal replacement by plant proteins in practical diets for juvenile gibel carp (*Carassius auratus gibelio*). Aquac. Nutr..

[41] Khosravi S., Bui H.T.D., Rahimnejad S., Herault M., Fournier V., Jeong J.B., Lee K.J. (2015). Effect of dietary hydrolysate supplementation on growth performance, non-specific immune response and disease resistance of olive flounder (*Paralichthys olivaceus*) challenged with Edwardsiella tarda. Aquac. Nutr..

[42] Zawistowski J., Kopec A., Kitts D.D. (2009). Effects of a black rice extract (*Oryza sativa* L. indica) on cholesterol levels and plasma lipid parameters in Wistar Kyoto rats. J. Funct. Foods.

[43] Reinhart K.M., Talati R., White C.M., Coleman C.I. (2009). The impact of garlic on lipid parameters: A systematic review and meta-analysis. Nutr. Res. Rev..

[44] Song Z.D., Li H.Y., Wang J.Y., Li P.Y., Sun Y.Z., Zhang L.M. (2014). Effects of fishmeal replacement with soy protein hydrolysates on growth performance, blood biochemistry, gastrointestinal digestion and muscle composition of juvenile starry flounder (*Platichthys stellatus*). Aquaculture.

[45] Ji K., Liang H.L., Ren M.C., Ge X.P., Mi H.F., Pan L.K., Yu H. (2020). The immunoreaction and antioxidant capacity of juvenile blunt snout bream (*Megalobrama amblycephala*) involves the PI3K/Akt/Nrf2 and NF-κB signal pathways in response to dietary methionine levels. Fish Shellfish Immunol..

[46] Zheng Q.M., Wen X.B., Han C.Y., Li H.B., Xie X.H. (2012). Effect of replacing soybean meal with cottonseed meal on growth, hematology, antioxidant enzymes activity and expression for juvenile grass carp, *Ctenopharyngodon idellus*. Fish Physiol. Biochem..

[47] Gaweł S., Wardas M., Niedworok E., Wardas P. (2004). Malondialdehyde (MDA) as a lipid peroxidation marker. Wiad. Lek..

[48] Yuan X.Y., Liu W.B., Wang C.C., Huang Y.Y., Dai Y.J., Cheng H.H., Jiang G.Z. (2020). Evaluation of antioxidant capacity and immunomodulatory effects of cottonseed meal protein hydrolysate and its derivative peptides for hepatocytes of blunt snout bream (*Megalobrama amblycephala*). Fish Shellfish Immunol..

[49] Gao D.D., Cao Y.S., Li H.X. (2010). Antioxidant activity of peptide fractions derived from cottonseed protein hydrolysate. J. Sci. Food Agric..

[50] Song W.G., Kong X.Z., Hua Y.F., Li X.F., Zhang C.M., Chen Y.M. (2020). Antioxidant and antibacterial activity and in vitro digestion stability of cottonseed protein hydrolysates. LWT.

[51] Bu X.Y., Chen A.J., Lian X.Q., Chen F.Y., Zhang Y., Muhammad I., Ge X.P., Yang Y.H. (2017). An evaluation of replacing fish meal with cottonseed meal in the diet of juvenile Ussuri catfish *Pseudobagrus ussuriensis*: Growth, antioxidant capacity, nonspecific immunity and resistance to *Aeromonas hydrophila*. Aquaculture.

[52] Boboev A., Hasanov A., Yotova L., Hasanov H. (2012). Antioxidant activity of peptides obtained from wheat and cottonseed proteins. Bulg. J. Agric. Sci..

[53] Tak P.P., Firestein G.S. (2001). NF-κB: A key role in inflammatory diseases. J. Clin. Investig..

[54] Li C.W., Li L.L., Chen S., Zhang J.X., Lu W.L. (2020). Antioxidant nanotherapies for the treatment of inflammatory diseases. Front. Bioeng. Biotechnol..

[55] Liu H., Dong X.H., Tan B.P., Du T., Zhang S., Yang Y.Z., Chi S.Y., Yang Q.H., Liu H.Y. (2020). Effects of fish meal replacement by low-gossypol cottonseed meal on growth performance, digestive enzyme activity, intestine histology and inflammatory gene expression of silver sillago (*Sillago sihama Forsskál*)(1775). Aquac. Nutr..

[56] Association of Official Analytical Chemists (1998). Official Methods of Analysis of the Association of Official Analytical Chemists.

[57] Huang D.Y., Liang H.L., Ge X.P., Zhu J., Li S.L., Wang Y.L., Ren M.C., Chen X.L. (2022). Effects of Dietary Lysine Levels on Growth Performance and Glycolipid Metabolism via the AKT/FoxO1 Pathway in Juvenile Largemouth Bass, *Micropterus salmoides*. Aquac. Nutr..

[58] Pfaffl M.W. (2001). A new mathematical model for relative quantification in real-time RT–PCR. Nucleic Acids Res..

[59] Zhao L.L., Liang J., Chen F.K., Tang X.H., Liao L., Liu Q., Luo J., Du Z.J., Li Z.Q., Luo W. (2021). High carbohydrate diet induced endoplasmic reticulum stress and oxidative stress, promoted inflammation and apoptosis, impaired intestinal barrier of juvenile largemouth bass *(Micropterus salmoides*). Fish Shellfish Immunol..

[60] Yu H., Liang H.L., Ge X.P., Zhu J., Wang Y.L., Ren M.C., Chen X.R. (2022). Dietary chlorella (*Chlorella vulgaris*) supplementation effectively improves body color, alleviates muscle inflammation and inhibits apoptosis in largemouth bass (*Micropterus salmoides*). Fish Shellfish Immunol..

[61] Gu J.Z., Liang H.L., Ge X.P., Xia D., Pan L.K., Mi H.F., Ren M.C. (2022). A study of the potential effect of yellow mealworm (*Tenebrio molitor*) substitution for fish meal on growth, immune and antioxidant capacity in juvenile largemouth bass (*Micropterus salmoides*). Fish Shellfish Immunol..

[62] Yang P., Wang W.Q., Chi S.Y., Mai K.S., Song F., Wang L. (2020). Effects of dietary lysine on regulating GH-IGF system, intermediate metabolism and immune response in largemouth bass (*Micropterus salmoides*). Aquac. Rep..

